# Dopamine disruption increases cleanerfish cooperative investment in novel client partners

**DOI:** 10.1098/rsos.160609

**Published:** 2017-05-03

**Authors:** Marta C. Soares, Teresa P. Santos, João P. M. Messias

**Affiliations:** CIBIO, Centro de Investigação em Biodiversidade e Recursos Genéticos, Universidade do Porto, Vairão, Portugal

**Keywords:** social familiarization, dopamine, cleanerfish, tactile stimulation, reward

## Abstract

Social familiarization is a process of gaining knowledge that results from direct or indirect participation in social events. Cooperative exchanges are thought to be conditional upon familiarity with others. Indeed, individuals seem to prefer to engage with those that have previously interacted with them, which are more accurate predictors of reward than novel partners. On the other hand, highly social animals do seek novelty. Truth is that the physiological bases underlying how familiarity and novelty may affect cooperative decision-making are still rather obscure. Here, we provide the first experimental evidence that the level of the dopaminergic influence in cooperative exchanges is constrained to mechanisms of social familiarization and novelty in a cleanerfish, *Labroides dimidiatus*. Cleaners were tested against familiar and novel *Ctenochaetus striatus* surgeonfish (a common client species) in laboratorial conditions, and were found to spend more time providing physical contact (also referred to as tactile stimulation) to familiar fish clients. Cleaners use tactile stimulation as a way to reduce the risk of a non-rewarding outcome, a behavioural response that is even more pronounced when blocking dopamine (DA) D1 receptors. We discovered that the influence of DA disruption on cleaners' provision of physical contact was dependent on the level of familiarity with its partner, being highly exacerbated whenever the client is novel, and unnoticed when dealing with a familiar one. Our findings demonstrate that DA mediation influences the valuation of partner stimuli and the enhancing investment in novel partners, mechanisms that are similar to other vertebrates, including humans.

## 1. Introduction

Dopamine (DA) is a neurotransmitter crucially involved in animals’ social cognition and decision-making [1,2], and it is mostly connected to reward, risk assessment and behaviour reinforcement [3,4]. Paired with a specific stimuli or action, DA release starts by signalling the delivery of an unexpected outcome (appetitive or aversive) [5,6]. Later, by repeating encounters, individuals learn to associate the outcome with the preceding stimuli, while DA response shifts from responding to the outcome to an earlier event-predicting stimuli or action. Thus, DA release works as a teaching signal that helps to predict a future outcome, based on previously learned associations or familiarization [6–9]. Familiarity then contributes to decreasing the subjective uncertainty and to increasing the predictor's accuracy concerning the outcome, which can be preponderant for deciding between different courses of action available and levels of investment.

On the other hand, animals do tend to seek out novelty. These novel stimuli have been reported to excite DA neurons [10,11] as well as raising signals in brain regions that receive dopaminergic input [12,13]. Thus, the increase of DA levels in response to novelty seems to be the mechanism that prompts animals to go exploring in search of reward. Is it possible that enhancing DA signalling will produce strong bias favouring choice preferences towards novel situations? In a recent study, Costa *et al*. [14] administered systemic injections of saline or GBR-12909, a selective DA transporter inhibitor, to male rhesus monkeys (*Macaca mullata*) and assessed their novelty-seeking behaviour during a probabilistic decision-making task. The authors found that dopaminergic blockage increased the monkeys' preference for novel options; however, the disruption did not influence the rate at which the monkeys learned to select predictive reward cues or their tendency to exploit that knowledge [14]. Thus, there is some evidence of a DA-dependent framework, modulating animals’ response to novelty and promoting exploratory behaviour without necessarily removing the importance of previously acquired social information (familiarity).

Among social species that are found to engage in cooperative behaviour, the case of the Indo-Pacific cleaner wrasse, *Labroides dimidiatus*, is a great example of a system in which individuals are faced with interacting with a wide set of other individual fish that may vary in familiarity or novelty. Cleaner fish inspect the surface, gills and sometimes the mouth of ‘client’ reef fish, eating ectoparasites, mucus, scales and dead or infected tissue [15], and may engage in hundreds of interactions per day [16]. And while the overall framework of the cleaning mutualisms involves repeated iterations with multiple partners (referred to as clients) that have asymmetrical options of behaving cooperatively or to defect (for review, see [17]), some are bound to be more familiar than others. Indeed, cleaners have been found to discriminate between familiar and non-familiar clients, choosing to spend more time near those they could recognize [18]. Moreover, cleaners are known to flexibly adjust cleaning service in accordance with client category, which they learn to distinguish (predators–non-predators, residents–visitors [17]), varying between cooperating (by removing ectoparasites) or defecting (by eating fish mucus). Interestingly, cleaner wrasses prefer to eat clients' mucus, which constitutes a defection (referred to as cheating), as it is costly for clients, and is thus responsible for multiple conflicts [19]. For instance, for the majority of non-predatory clients, defecting is not an option; in response, these may terminate an interaction (sanction), chase the cleaner (punishment) and exert partner choice (cleaner switching) [17]. In response, cleaner wrasses flexibly adjust their cheating frequency and may also improve their service investment by providing a form of physical contact to clients (known as tactile stimulation or massages), touching them with their pelvic fins [20]. Cleaner wrasses use tactile stimulation in several different contexts, but mostly in cases of uncertainty about the interaction outcome: to reconcile after a cheating event, to prolong interactions with clients about to leave the cleaning station, and as a pre-conflict management strategy with predators [21,22]. Clients benefit from receiving tactile stimulation as it lowers baseline and acute stress levels [23].

Recent evidence has shown that the DA system is significantly linked to decisions of cleaner wrasses during interactions with clients, particularly those concerning the provision of tactile stimulation and the function of the D1 receptor [24,25]. Messias *et al*. [25] found that the inhibition of DA receptors appeared to cause cleaners to behave as if clients were permanently in dispute about the value of being serviced, due to a putative increase of sensitivity to negative or uncertain outcomes (risk aversion). Moreover, by blocking the DA receptors (D1- and D2-like receptors), cleaner wrasses displayed higher willingness to engage in interactions and to increase tactile stimulation provision to clients, behaviours that cleaners usually use to increase the probability of receiving the expected reward by preventing the premature termination of the interaction or the chasing on behalf of the client. However, it reduced their overall food intake (cleaning behaviour). It is not clear whether DA blockage was selectively disrupting cleaners' ability to predict clients’ behaviour (i.e. cleaners were appraising all clients as novel), or how the manipulation of clients’ familiarity would interfere with the modulation of DA. To test for this, we administrated a D1 receptor agonist (D1a—SKF38393), an antagonist (D1an—SCH23390), as well as a control (Saline) to cleaners introduced to familiar and to novel (non-familiar) clients. Considering the effect of DA blockage on novelty-driven behaviour [14], we predicted stronger effects of DA disruption on cleaner behavioural engagement and tactile stimulation provision when dealing with novel clients. However, referring to DA impact on learning [24], we could also expect an effect of the DA agonist on cleaners’ approach to familiar clients.

## 2. Material and methods

### 2.1. Study species and housing conditions

Experiments were conducted between November and December 2014 at U.C. Berkeley Gump Research Station in Mo'orea, French Polynesia. Permission and licence to conduct animal collections and laboratorial experimentation were provided by the University of California Animal Ethics committee. All fish were captured from the surrounding reefs by SCUBA divers, and released after the end of the experiments. Before the beginning of the experiments, all fish were given a period of 9–15 days to acclimatize to the captive conditions (cleaners and clients, respectively). Ten *L. dimidiatus* cleaner wrasses were placed in individual aquaria (90 × 40 × 50 cm) and small PVC pipes (10–15 cm long, 2.5 cm diameter) were included as shelters for the cleanerfish. *Ctenochaetus striatus* surgeonfish were chosen as clients due to their abundance and status as frequent clients at cleaning stations. Eight individuals were haphazardly chosen, captured and kept in pairs in round holding tanks (four smaller tanks with 120 cm diameter, approx. 1000 l and one large tank with 140 cm diameter, approx. 1350 l whenever clients needed to be separated), where several large PVC pipes (20 cm long, 10 cm diameter) were placed as shelters. Both the aquaria and tanks had an individual constant flow of seawater directly pumped from the sea.

### 2.2. Experimental design

Experiments were planned for each cleaner to have only one familiar client. To create familiarity, the respective client was presented to the cleaner for 5 consecutive days, for a total of 14 h divided in 3 h sessions (between 09.30 and 18.30 h), except in the first day in which a 2 h session was conducted. As for the non-familiar clients, these were solely introduced to the cleaner during the appointed experimental trial (see electronic supplementary material, table S1). Cleaners were weighed before the onset of the experiment so that injection volumes could be adjusted to body weight. The following compound treatments were used: saline solution as control (0.9% NaCl); a selective D1 agonist SKF-38393 (D047, Sigma); D1 antagonist SCH-23390 (D054, Sigma). Injection volumes were always 15 µl per gram of estimated body weight (gbw). This process never exceeded 3 min. Dosages applied were based on previous studies: 5.0 µg gbw^−1^ of SKF-38393 [24–28] and 0.5 µg gbw^−1^ of SCH-23390 [24,25,29,30].

**Figure 1.**
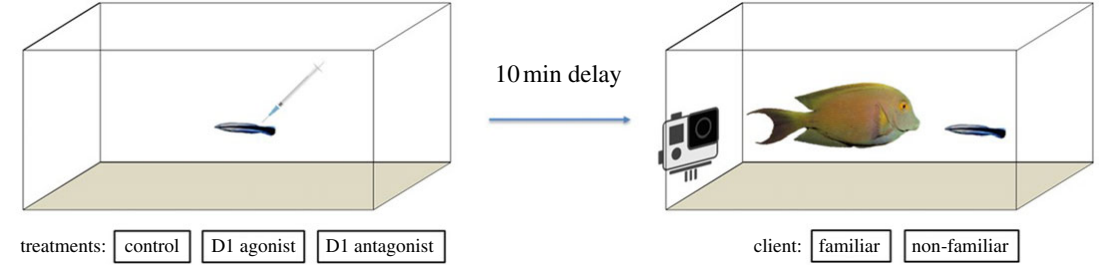
The experimental set-up (lateral view): (*a*) cleaner wrasses *Labroides dimidiatus*, were previously injected with a dopamine D1 receptor agonist (SKF-38393), D1 antagonist (SCH-23390) or a saline control before each test; (*b*) following a 10 min delay, cleaners were introduced to a familiar or a non-familiar (novel) client.

After the injection of the respective compound, there was a 10 min delay before the client was placed in the cleaner wrasse aquarium for the beginning of the trial, which lasted for 30 min (figure 1). All cleaner wrasses interacted with familiar and non-familiar clients under the effects of the injection of each compound, with a total of six trials for each cleaner wrasse, and injected cleaners were given at least 48 h of rest between injections. The order of these trials (familiar or non-familiar context versus compound treatment administration) was randomly chosen between trial sessions. Experiments were conducted on alternate days, and each day of experiments was divided in three recording sessions (each video coded, to be ‘blindly’ analysed later), between 08.30 and 13.00 h. Thus, all cleaners were injected on the same day, but each one only once per day.

### 2.3. Behaviour observations

The following measurements were noted for each interaction filmed on video: (i) who initiated the interaction—clients were scored as the ones soliciting an interaction if they posed before the cleaner touched them, otherwise the cleaner was scored as the one initiating; (ii) duration (in seconds) of inspection towards each client; (iii) the number and duration of tactile stimulation events provided to each client; (iv) number of jolts performed by clients; and (v) frequency of punishments towards cleaners.

### 2.4. Statistical analysis

Measures of cleaner wrasse behaviour towards clients were divided into three categories: (i) measures of likelihood to engage in cleaning behaviour (motivation to interact); (ii) measures of interactive investment (provision of tactile stimulation) and (iii) a measure of cleaner wrasse cheating levels (client jolt rate). The likelihood to engage with a client was measured by: (1) the proportion of cleaning interactions (calculated as the total number of cleaning interactions/total number of solicitations) and (2) the mean duration of inspection (total time of interaction/total number of interactions). Measures of investment were calculated as: (1) the proportion of interactions in which tactile stimulation was used by cleaners (frequency of interactions where tactile stimulation occurred/total number of interactions) and (2) the proportion of time cleaners spent providing tactile stimulation to a client (total tactile stimulation duration/total interaction duration). Finally, the measure of cleaners’ cheating levels was calculated using the frequency of jolts per 100 s of inspection.

To examine the influence of familiarity and DA treatment on cleaner wrasses' behaviour we used a Linear Nested Mixed Model, with context (two levels: familiar and non-familiar) and treatment (three levels: D1 agonist, D1 antagonist and saline) as fixed factors, but with treatment nested within context, while controlling for cleaner identity and trial sequence (as random factors). Planned comparisons between treatments were examined by using Bonferroni's pairwise comparisons. All tests were two-tailed and were done in IBM SPSS Statistics, v. 22.

## 3. Results

**Figure 2.**
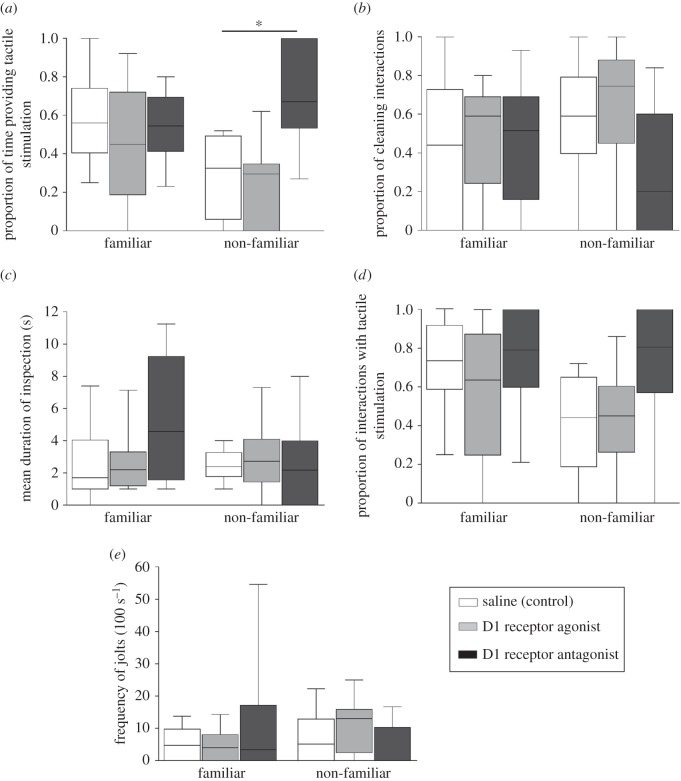
The effect of exogenous injections of dopaminergic compounds, the D1 agonist (D1a), antagonist (D1an) and saline (control), on: (*a*) the proportion of time cleaners *Labroides dimidiatus* spent providing tactile stimulation (total tactile stimulation duration/total interaction duration), (*b*) the proportion of cleaning interactions (calculated as the total number of cleaning interactions/total number of solicitations), (*c*) the mean duration of inspection (total time of interaction/total number of interactions), (*d*) the proportion of interactions in which tactile stimulation was used by cleaners (frequency of interactions where tactile stimulation occurred/total number of interactions) and (*e*) the frequency of jolts per 100 s of inspection; whenever introduced to: familiar and non-familiar client, *Ctenochaetus striatus*. Medians, interquartile ranges and maximum and minimum are shown. The significant value is shown above bars, * < 0.01, and refers to a Bonferroni pairwise comparison against saline (control). A total sample size of 10 individuals was used.

The proportion of time providing tactile stimulation varied according to the model interaction between familiarity context and DA treatment (Linear Nested Mixed Model *F* = 6.372; *p* = 0.002; figure 2*a*; electronic supplementary material, table S2), while no interaction was found for any of the other behavioural measures considered (Linear Nested Mixed Models: all *p* > 0.050; figure 2*b–e*; electronic supplementary material, tables S3–S6). In a context of non-familiarity, cleaners treated with the D1 antagonist spent a greater proportion of inspection time providing tactile stimulation compared with control (Bonferroni's pairwise comparison: *p* = 0.007, figure 2*a*), while no significant effects were found with the D1 agonist (Bonferroni's pairwise comparison *p* = 0.951, figure 2*a*). Otherwise, dopaminergic treatment revealed to be inefficient in a context of familiarity (Bonferroni's pairwise comparisons, proportion of inspection time providing tactile stimulation, D1 agonist versus saline: *p* = 0.625 and D1 antagonist versus saline: *p* = 0.965, figure 2*a*).

## 4. Discussion

To gain benefits, animals involved in cooperative exchanges are thought to make use of the predictive structure of the social environment, by trusting previously learned (familiar) signals that account for the occurrence of reward, while distinguishing signals that carry higher risk but potentially bring an absence of benefits [31]. Animals learn not only to react to stimuli that favour reward (higher food intake) but also to adjust their behavioural responses when stimuli are associated with bad outcomes or to higher propensity for reward omission [32]. However, animals' propensity to explore novel environments and partners is also key to establish new partnerships and to gain new sources of reward. Cleaners are able to interact with multiple partners (belonging to several different species) during a single day [16]. The iterated framework of cleaners' complex social environment gives space to a dual motivational drive: one focusing on the importance of predictability and reward gain based on familiar clients, and the other promoting pro-sociality and the exploration of novel partners. Indeed, novelty may be rewarding in itself, which is a response to DA signalling [14]. In the case of cleaner wrasses, their reward (access to any given client) begins to be secured as they approach and get in direct physical contact with clients [21]. Truly, physical contact (tactile stimulation) is a mechanism used to reduce the risk of a non-rewarding outcome (such as an early termination of an interaction by a client) that is more often exhibited when blocking the D1 receptors [25].

Here, we probed for the implications of DA shifts to cleaners’ behaviour in response to a familiar partner compared with a stranger. While cleaners appeared to generally provide more tactile stimulation to familiar clients than to novel ones, irrespectively of DA treatment, the effect of blockage solely scaled when these dealt with novel clients. The blockage effect was expected considering previous evidence on humans and other primates, which suggested that DA reuptake capacity contributed to exacerbated novelty seeking [14]. Interestingly, this effect strongly suggests that cleaners value novelty more than the so-called costs associated with unpredictability, and that this boldness is modulated by DA shifts. Nevertheless, cleaners behavioural response to novelty (i.e. the provision of tactile stimulation) due to DA disruption was not affecting the tactile stimulation given to predictable cues (familiar clients). This seems to contrast with previous observations in the wild, where cleaners were observed to provide more tactile stimulation to novel clients so as to build up new relationships [33]. However, these results could have been significantly influenced by cleaners' putative increase of stress in response to capture and translocation, a relevant variable influencing behavioural change [34]. Here, control (saline infused) cleaners provided more tactile stimulation to previously acquainted clients as it is, perhaps, within the category of familiar clients that they learn to discriminate high, medium and low value customers, expecting certain amounts of reward per visit.

The absence of effects observed in the proportion of cleaning interactions and the proportion of interactions with tactile stimulation provision, for both D1 agonist and antagonist treatments were not entirely surprising considering the overall number of interactions recorded, which were predictably lower in controlled conditions compared with those reported in the wild [25]. The mean duration of inspections was relatively constant between contexts and treatments, except for the visual effect of the D1 antagonist on familiar clients: while lacking statistical significance, cleaners seemed to be spending more time inspecting these clients under reduction of normal DA signalling (figure 2*c*). Finally, the frequency of client jolts did not seem to vary with changes in DA tone, similarly to what was reported by Messias *et al*. [25].

Our data show, for the first time, that DA mechanisms modulate cleaners' response to novel partners, particularly in shifting the amount of tactile stimulation provided to clients. The effects of DA inhibition were only effective when dealing with novel partners (i.e. in the absence of any previously acquired social information related to those partners), but otherwise did not seem to affect cleaner response to familiar clients (predictive cues). Our results further demonstrate that DA mediation underlies the valuation of partner stimuli, enhancing response and investment to novel partners, mechanisms that are similar to other vertebrates, including humans [12,14].

## Supplementary Material

Suplementary material

## Data accessibility

Supporting behavioural data can be accessed at Dryad at http://dx.doi.org/10.5061/dryad.j8r34 [35].
