# Supplementary material for: Dopamine disruption increases cleanerfish cooperative investment in novel client partners
Source: R Soc Open Sci. 2017 May 3;4(5):160609. doi: 10.1098/rsos.160609 (PMC5451786; doi:10.1098/rsos.160609)
Supplement: Suplementary material [file rsos160609supp1.docx]

**Supplementary Material**

Table S1. Context (Familiarity (F) and non-familiarity (N)), compound (D1 antagonist, D1 agonist and saline) and session correspondence between cleaners and clients’ identity (Cs).

| Cleaner | Client | Context | Compound | Session |
| --- | --- | --- | --- | --- |
| 1 | C5 | N | D1 Ant | 5 |
| 1 | C1 | F | Saline | 11 |
| 1 | C1 | F | D1 Agon | 17 |
| 1 | C7 | N | D1 Agon | 20 |
| 1 | C8 | N | Saline | 22 |
| 1 | C1 | F | D1Ant | 25 |
| 2 | C5 | N | D1 Ant | 3 |
| 2 | C6 | N | Saline | 8 |
| 2 | C1 | F | D1 Ant | 12 |
| 2 | C7 | N | D1 Agon | 15 |
| 2 | C1 | F | Saline | 20 |
| 2 | C1 | F | D1 Agon | 23 |
| 3 | C8 | N | Saline | 2 |
| 3 | C6 | N | D1 Agon | 5 |
| 3 | C1 | F | Saline | 9 |
| 3 | C7 | N | D1 Ant | 10 |
| 3 | C1 | F | D1 Agon | 13 |
| 3 | C1 | F | D1 Ant | 16 |
| 4 | C2 | F | D1 Agon | 1 |
| 4 | C2 | F | D1 Ant | 4 |
| 4 | C6 | N | D1 Agon | 10 |
| 4 | C2 | F | Saline | 17 |
| 4 | C7 | N | D1 Ant | 21 |
| 4 | C8 | N | Saline | 23 |
| 5 | C6 | N | Saline | 3 |
| 5 | C2 | F | D1 Ant | 7 |
| 5 | C1 | N | D1 Agon | 14 |
| 5 | C2 | F | Saline | 16 |
| 5 | C8 | N | D1 Ant | 19 |
| 5 | C2 | F | D1 Agon | 23 |
| 6 | C8 | N | Saline | 5 |
| 6 | C2 | F | D1 Agon | 12 |
| 6 | C5 | N | D1 Ant | 15 |
| 6 | C4 | N | D1 Agon | 16 |
| 6 | C2 | F | D1 Ant | 20 |
| 6 | C2 | F | Saline | 24 |
| 7 | C3 | F | D1 Ant | 1 |
| 7 | C3 | F | D1 Agon | 7 |
| 7 | C8 | N | D1 Ant | 10 |
| 7 | C3 | F | Saline | 17 |
| 7 | C1 | N | D1 Agon | 21 |
| 7 | C7 | N | Saline | 22 |
| 8 | C7 | N | D1 Ant | 3 |
| 8 | C3 | F | D1 Ant | 4 |
| 8 | C2 | N | D1 Agon | 14 |
| 8 | C6 | N | Saline | 16 |
| 8 | C3 | F | Saline | 20 |
| 8 | C3 | F | D1 Agon | 23 |
| 9 | C3 | F | D1 Ant | 2 |
| 9 | C6 | N | Saline | 6 |
| 9 | C3 | F | Saline | 12 |
| 9 | C8 | N | D1 Agon | 14 |
| 9 | C7 | N | D1 Ant | 18 |
| 9 | C3 | F | D1 Agon | 21 |
| 10 | C4 | F | D1 Agon | 1 |
| 10 | C4 | F | Saline | 6 |
| 10 | C2 | N | D1 Agon | 9 |
| 10 | C5 | N | D1 Ant | 10 |
| 10 | C4 | F | D1 Ant | 13 |
| 10 | C7 | N | Saline | 24 |

Table S2. Statistical results of multifactor (nested) linear mixed model. Two factors are fixed; Treatment: D1 agonist; D1 antagonist; saline and Context: familiar or non-familiar. The effect of treatment is nested within context. Two factors are random: Treatment sequence and client identity. The proportion of time cleaners spent providing tactile stimulation to a client is the dependent variable. P < 0.05 values are in bold; * significant term interpreted in the text.

| Source | Numerator DF | Denominator DF | *F* | *P* |
| --- | --- | --- | --- | --- |
|  |  |  |  |  |
| Intercept | 1 | 1.761 | 82.086 | 0.018 |
| Treatment (context) | 5 | 16.253 | 6.372 | **0.002*** |
|  |  |  |  |  |

Table S3. Statistical results of multifactor (nested) linear mixed model. Two factors are fixed; Treatment: D1 agonist; D1 antagonist; saline and Context: familiar or non-familiar. The effect of treatment is nested within context. Two factors are random: Treatment sequence and client identity. The proportion of cleaning interactions is the dependent variable. Logarithmical transformation was used. P < 0.05 values are in bold; * significant term interpreted in the text.

| Source | Numerator DF | Denominator DF | *F* | *P* |
| --- | --- | --- | --- | --- |
|  |  |  |  |  |
| Intercept | 1 | 1.410 | 59.859 | 0.04 |
| Treatment (context) | 5 | 14.001 | 1.201 | 0.358 |
|  |  |  |  |  |

Table S4. Statistical results of multifactor (nested) linear mixed model. Two factors are fixed; Treatment: D1 agonist; D1 antagonist; saline and Context: familiar or non-familiar. The effect of treatment is nested within context. Two factors are random: Treatment sequence and client identity. The mean duration of inspection is the dependent variable. Logarithmical transformation was used. P < 0.05 values are in bold; * significant term interpreted in the text.

| Source | Numerator DF | Denominator DF | *F* | *P* |
| --- | --- | --- | --- | --- |
|  |  |  |  |  |
| Intercept | 1 | 5.101 | 99.148 | < 0.0001 |
| Treatment (context) | 5 | 11.072 | 1.116 | 0.406 |
|  |  |  |  |  |

Table S5. Statistical results of multifactor (nested) linear mixed model. Two factors are fixed; Treatment: D1 agonist; D1 antagonist; saline and Context: familiar or non-familiar. The effect of treatment is nested within context. Two factors are random: Treatment sequence and client identity. The proportion of interactions in which tactile stimulation was used by cleaners is the dependent variable. P < 0.05 values are in bold; * significant term interpreted in the text.

| Source | Numerator DF | Denominator DF | *F* | *P* |
| --- | --- | --- | --- | --- |
|  |  |  |  |  |
| Intercept | 1 | 3.739 | 177.584 | < 0.0001 |
| Treatment (context) | 5 | 14.837 | 2.845 | 0.054 |
|  |  |  |  |  |

Table S6. Statistical results of multifactor (nested) linear mixed model. Two factors are fixed; Treatment: D1 agonist; D1 antagonist; saline and Context: familiar or non-familiar. The effect of treatment is nested within context. Two factors are random: Treatment sequence and client identity. The frequency of jolts per 100 seconds is the dependent variable. Square root transformation was used. P < 0.05 values are in bold; * significant term interpreted in the text.

| Source | Numerator DF | Denominator DF | *F* | *P* |
| --- | --- | --- | --- | --- |
|  |  |  |  |  |
| Intercept | 1 | 2.008 | 30.202 | 0.031 |
| Treatment (context) | 5 | 20.067 | 0.482 | 0.785 |
|  |  |  |  |  |
